# Evaluation of Serum Lactate Dehydrogenase and Electrolyte Levels Among Preeclamptic Women at the University of Gondar Comprehensive Specialized Hospital, Northwest, Ethiopia: A Comparative Cross‐Sectional Study

**DOI:** 10.1155/jp/9970060

**Published:** 2026-02-12

**Authors:** Endeshaw Yitayew Tamirie, Hiwot Tezera Endale, Muluken Fekadie Zerihun, Fikadu Seyoum Tola, Mohammed Jemal, Meseret Derbew Molla

**Affiliations:** ^1^ Department of Biochemistry, College of Medicine and Health Sciences, Ambo University, Ambo, Ethiopia, ambou.edu.et; ^2^ Department of Medical Biochemistry, School of Medicine, College of Medicine and Health Sciences University of Gondar, Gondar, Ethiopia; ^3^ Department of Medical Biochemistry, School of Medicine, College of Medicine and Health Sciences, Addis Ababa University, Addis Ababa, Ethiopia, aau.edu.et; ^4^ Department of Biomedical Science, School of Medicine, Debre Markos University, Debre Markos, Ethiopia, dmu.edu.et; ^5^ College of Medicine and Public Health, Flinders Health and Medical Research Institute, Flinders University, Adelaide, South Australia, Australia, flinders.edu.au

**Keywords:** electrolytes, lactate dehydrogenase, normotensive, preeclampsia

## Abstract

**Background:**

Serum lactate dehydrogenase is a sensitive marker of hypoxia and cellular damage/death in preeclampsia due to vascular endothelial dysfunction. Evaluation of serum electrolytes can also indicate the severity of preeclampsia since it is a vascular endothelial disorder. However, in Ethiopia, there is a lack of data regarding the serum levels of lactate dehydrogenase and electrolytes among preeclamptic patients in comparison with apparently healthy normotensive pregnant women.

**Method:**

A hospital‐based comparative cross‐sectional study was conducted with 128 participants (64 preeclamptic women and 64 apparently healthy normotensive pregnant women) from October 20, 2021 to January 3, 2022. Preeclamptic women were further classified into 32 without severe features and 32 with severe features. Blood samples (5 mL) were collected, and a Beckman Coulter 700 AU chemistry analyzer was used to measure serum LDH and electrolyte levels. The data were analyzed using SPSS version 25. One‐way ANOVA was run to examine the mean variations, and the receiver operator characteristic curve was used to determine the potential diagnostic value for preeclampsia. A *p* value < 0.05 was considered statistically significant.

**Result:**

Preeclamptic women showed a statistically significant elevation of LDH (*p* < 0.001) when compared with apparently healthy normotensive pregnant women. There was a substantial decrement in serum magnesium (*p* < 0.001), calcium (*p* < 0.001), and potassium (*p* = 0.029) in preeclamptic women than apparently healthy normotensive pregnant women. There was also a significant elevation of LDH (*p* < 0.001) but a reduction of calcium (*p* < 0.001) and potassium (*p* = 0.021) in preeclampsia with severe features than preeclampsia without severe features. Serum LDH detected preeclampsia with excellent accuracy (97.5%) at ≥ 350 U/L. Serum magnesium, calcium, and potassium demonstrated diagnostic accuracies of 69.5%, 82.3%, and 61%, respectively, for identifying preeclampsia, with optimal cutoff values of ≤ 1.505 mg/dl for magnesium, ≤ 8.65 mg/dl and 8.75 mg/dl for calcium, and 3.5 mmol/l and 3.75 mmol/l for potassium.

**Conclusion:**

Serum LDH was significantly increased in the preeclamptic group compared with normotensive controls. There were significantly decreased levels of serum electrolytes (magnesium, calcium, and potassium) in preeclamptic women. Therefore, it is better to focus on the measurement of serum LDH and electrolytes for early detection and effective management of preeclampsia.

## 1. Introduction

Preeclampsia is the new‐onset of hypertension (blood pressure of ≥ 140/90 mmHg) at or after 20 weeks of gestation accompanied by proteinuria or any signs of end‐organ damage, and thrombocytopenia with a platelet count below 150,000/dl. Proteinuria is defined as the presence of 300 mg or more of protein in a 24‐h urine collection or a urine dipstick protein reading of +1 [1]. It may result from an imbalance between pro‐angiogenic and anti‐angiogenic factors, leading to podocyte injury and disruption of the glomerular filtration barrier [2]. Preeclampsia is the second leading cause of maternal mortality globally, causing around 76,000 maternal and 500,000 perinatal deaths each year [3]. Its global incidence is about 4.6%, ranging from 1.0% in the Eastern Mediterranean to 5.6% in Africa [4]. In Ethiopia, where maternal mortality remains high, preeclampsia accounts for roughly 10% of maternal deaths, with a pooled prevalence of 4.74% [5].

Although the exact etiology is not known, preeclampsia is a multifactorial disorder in which abnormal placentation plays a central role. In normal pregnancy, cytotrophoblasts (CTBs) invade the uterine wall and remodel spiral arteries into low‐resistance vessels that ensure adequate placental blood flow [6]. In preeclampsia, this process is defective, characterized by shallow trophoblastic invasion and incomplete spiral artery remodeling, leading to persistent placental hypoxia [7]. Placental hypoxia impairs endothelium‐dependent vasorelaxation, reduces endothelial nitric oxide synthase (eNOS) activity, and increases oxidative stress through endothelin and NADPH oxidase pathways [8]. This, in turn, triggers the release of soluble factors into the maternal circulation, causing widespread vascular endothelial dysfunction, which is regarded as the hallmark of preeclampsia [3, 7].

Endothelial injury/dysfunction reduces perfusion to multiple maternal and fetal organs, leading to systemic hypoxia and impairing the function of vital organs such as the brain, liver, kidneys, and placenta. These processes contribute to serious maternal complications including placental abruption, hepatic failure, acute renal failure, neurological injury, and cardiovascular collapse [9]. Endothelial dysfunction and tissue hypoxia are also reflected in measurable biochemical alterations. Lactate dehydrogenase (LDH), an intracellular enzyme catalyzing the conversion of pyruvate to lactate, rises during hypoxia due to enhanced anaerobic glycolysis [10]. Its elevation in preeclampsia also reflects cellular injury and necrosis secondary to vascular endothelial damage [11]. However, most studies in Ethiopia have focused on prevalence and nonbiochemical risk factors, with limited attention to potential biomarkers such as LDH.

Serum electrolytes levels may also play a role in preeclampsia since it is a vascular endothelial disorder. A recent systemic review and meta‐analysis has shown lower serum magnesium levels in women with preeclampsia compared with normotensive pregnant controls [12]. Magnesium regulates vascular tone and neuromuscular function by acting as a natural calcium antagonist and vasodilator; its deficiency promotes calcium influx, vasoconstriction, and increased vascular reactivity [13]. Conversely, adequate calcium intake reduces vasoconstrictor responses and vascular hyperreactivity, key features of preeclampsia. The 2018 Cochrane Review supports calcium supplementation (≥ 1 g/day) to reduce preeclampsia risk, particularly in populations with low dietary calcium [14]. Both calcium and magnesium regulate vascular tone and blood pressure, whereas magnesium also enhances prostaglandin E synthesis, potassium‐mediated vasodilation, and sodium–potassium ATPase activity [13, 15, 16]. Disturbances in sodium and potassium homeostasis have also been implicated in preeclampsia. Although earlier studies found no clear benefit of salt restriction, emerging evidence suggests sodium may influence placental and vascular function, underscoring the need for individualized rather than uniform dietary recommendations [17]. Potassium, a key regulator of membrane potential and endothelial function, promotes vasodilation via nitric oxide synthesis and sodium–potassium ATPase activation [16]. Reduced potassium levels and an elevated sodium‐to‐potassium ratio are associated with vascular stiffness and hypertension, highlighting their potential relevance in preeclampsia [18]. Given that preeclampsia involves placental hypoxia‐induced endothelial dysfunction, assessment of serum parameters reflecting vascular injury—particularly LDH and electrolytes, may enhance the assessment of disease severity and risk of complications. Despite these, no study has been done in Ethiopia to assess these biochemical alterations.

### 1.1. Aim and Objectives

General objective:

This study is aimed at assessing serum LDH and electrolyte levels in preeclamptic patients and comparing them with apparently healthy normotensive pregnant controls at the University of Gondar Comprehensive Specialized Hospital, Northwest Ethiopia, 2021.

Specific objectives:

1. To compare mean serum LDH levels between preeclamptic women and apparently healthy normotensive pregnant women.
2. To compare mean serum electrolyte levels (calcium, magnesium, sodium, and potassium) between the two groups.
3. To evaluate the potential diagnostic value of LDH and electrolytes in identifying preeclampsia.

## 2. Materials and Methods

### 2.1. Study Setting and Period

This study was conducted among 64 preeclamptic women and 64 apparently healthy normotensive pregnant women at UGCSH. The University of Gondar is one of Ethiopia′s known medical universities, and its comprehensive specialized hospital is one of the major teaching hospitals in the country. On average, 300–400 pregnant women are booked for antenatal care services in the hospital every month. About 60–100 preeclamptic patients are getting services monthly, including those referred cases and newly diagnosed in the antenatal care clinic and labor triage. The hospital provides more than 10,000 mothers′ delivery services per year. The study was conducted from October 20, 2021 to January 3, 2022.

### 2.2. Study Design

A hospital‐based comparative cross‐sectional study was conducted at UGCSH.

### 2.3. Source and Study Population

Pregnant women with gestational age greater than 20 weeks, either with normal blood pressure or with preeclampsia, who attended the antenatal clinic at UGCSH, were included as controls and cases in our study, respectively.

### 2.4. Eligibility Criteria

#### 2.4.1. Inclusion Criteria

1. Preeclamptic group: preeclamptic patients visiting UGCSH during the study period.
2. Comparison group: apparently healthy normotensive pregnant women whose blood pressure is < 140/90 mmHg and gestational age > 20 weeks visiting the antenatal care unit of UGCSH during the study period.

#### 2.4.2. Exclusion Criteria

Women with chronic hypertension, chronic kidney disease, neuromuscular disorders, diabetes mellitus, cardiac disease, chronic liver disease, bone disorders, smokers, and alcohol consumers were excluded from both groups. Additionally, preeclamptic women who had already received magnesium sulfate treatment were excluded.

### 2.5. Study Variables

#### 2.5.1. Dependent Variables

The dependent variables for this study are serum LDH and serum electrolyte (calcium, magnesium, sodium, and potassium) levels.

#### 2.5.2. Independent Variables

The independent variables for this study are sociodemographic variables (age, occupation, residence, income level, and educational status), gestational age, trimester, gravidity, parity, symptoms (epigastric pain, headache, and blurred vision).

### 2.6. Operational Definitions

Preeclampsia without severe features (formerly called mild preeclampsia): New‐onset of gestational hypertension, defined as SBP ≥ 140 mmHg and/or DBP ≥ 90 mmHg measured at least two times 4 h apart at or after 20 weeks of gestation accompanied by new‐onset proteinuria without other various laboratory and systemic findings, which would qualify as preeclampsia with severe features [19].

Preeclampsia with severe features (formerly called severe preeclampsia): hypertension ≥ 160/110 mmHg, thrombocytopenia (platelet count < 100,00/dl), elevated liver function at least two times (ALT or AST ≥70 U/l), progressive renal insufficiency (serum creatinine ≥ 100 *μ*mol/l; 1.1 mg/dL), pulmonary edema, new‐onset cerebral or visual disturbances including severe headache, blurred vision and hyperreflexia [19].

### 2.7. Sample Size Determination and Sampling Procedure

#### 2.7.1. Sample Size Determination

The sample size was determined by using G∗ Power Version 3.1.9.4. G∗ Power is one of the software packages that helps to perform sample size calculations. As an input, G∗ Power requires selecting an appropriate test family (*t*‐test in this case) and specifying the alpha error probability, power (1‐*β* error probability), allocation ratio, and effect size. So, considering the assumption of comparison of two independent sample means, using a two‐tailed *t*‐test, significance level *α* = 0.05, power = 80*%*, effect size *d* = 0.5, and an allocation ratio of 1, a total of 128 (64 preeclamptic women and 64 apparently healthy normotensive pregnant women) samples were determined.

##### 2.7.1.1. Sampling Procedure

A consecutive convenience sampling technique was employed until the required sample size was achieved. Eligible women (gestational age > 20 weeks) attending the UGCSH antenatal clinic or labor triage during the study period were approached, and those who volunteered provided written informed consent.

### 2.8. Data Collection and Laboratory Analysis

Written informed consent was obtained from the study participants. Trained health professionals collected the data. A semistructured interviewer‐administered questionnaire was used for data collection. The questionnaire was developed after reviewing relevant literature and included information about the participants′ sociodemographic condition, obstetrics, medical status, and lifestyle [5, 11]. Each participant′s blood pressure was taken on the right hand in a sitting position every 4 hours using the digital automated blood pressure apparatus applying standard precautions. The measurements were taken after the participant rested for at least 5 min or 30 min for those who took hot drinks like coffee. The average blood pressure of each patient was obtained from three consecutive readings at 2–3 min intervals [20]. After 4 h, the average of three consecutive readings at 2–3 min intervals was recorded, and the mean of measurements was taken for analysis.

### 2.9. COVID‐19 Prevention Protocol

To minimize the risk of COVID‐19 transmission, data collectors used personal protective equipment and disinfected apparatus before each interview. Study participants were advised to wear face masks, sanitize their hands, and maintain social distancing during data collection.

### 2.10. Blood Sample Collection and Processing

After the study, participants were asked for their informed consent to be interviewed and to give a blood sample of about 5‐mL venous blood drawn aseptically through vein puncture and collected through a serum separator test tube by trained health professionals. The drawn blood specimen was allowed to stay for 30 min for clot formation and then centrifuged at 4000 revolutions per minute (rpm) for 5 min to separate the serum from the whole blood. The separated serum sample was transferred immediately to the laboratory for analysis or stored at 2°C–8°C until the analysis was done. A Beckman Coulter 700 AU chemistry analyzer measured serum LDH and electrolyte levels according to the manufacturer′s instructions. The normal reference values of biochemical parameters are shown in Table 1.

**Table 1:** Normal reference values of biochemical parameters [21].

**Biochemical test**	**Second trimester**	**Third trimester**
LDH (U/L)	80–447	82–524
Magnesium (mg/dl)	1.5–2.2	1.1–2.2
Calcium (mg/dl)	8.2–9	8.2–9.7
Sodium (meq/l)	129–148	130–148
Potassium (meq/l)	3.3–5	3.3–5.1

### 2.11. Data Processing and Analysis

The collected data were checked for completeness and entered into a computer using Epi‐Data Version 4.6 software and exported to SPSS Version 25 for analysis. Then, the comparisons between groups were performed by *t*‐tests for continuous variables and chi‐square tests for categorical variables. The Shapiro–Wilk normality test and visual inspection of the histogram, kurtosis, normal Q–Q plot, and box plot tests checked the normal distribution of data. Leven′s test was applied to check whether the data met the equal variance assumption or not. One‐way ANOVA was done to analyze variations of mean levels of LDH and electrolyte levels for normotensive pregnant, preeclampsia without severe features, and preeclampsia with severe features, and a *p* value of < 0.05 was declared statistically significant. Moreover, the receiver operator characteristic curve was used to calculate the sensitivity and specificity predictive values of each significantly correlated laboratory parameter with blood pressure. The area under the curve (AUC) was calculated to evaluate the predictive power. The ranges of AUC values and their corresponding classification quality are presented in Table 2. Each optimal cutoff point was assessed by calculating the maximum value of sensitivity + specificity – 1 (Youden index). A *p* value < 0.05 was considered to be statistically significant.

**Table 2:** AUC value ranges and the corresponding quality of classification [22].

**AUC value**	**Quality**
0.9–1.0	Excellent
0.8–0.9	Very good/considerable
0.7–0.8	Good/fair
0.6–0.7	Poor
< 0.6	Fail

## 3. Result

### 3.1. Sociodemographic Characteristics

A total of 128 participants (64 preeclamptic women and gestational age and age‐matched 64 apparently healthy normotensive pregnant women) were enrolled in the study. The preeclamptic women were further classified into preeclampsia with and without severe features (*n* = 32 for each) to study the relationship of serum parameters with disease severity. The minimum age was 19 years in both groups, whereas the maximum age was 38 years for preeclamptic women and 36 years for apparently healthy normotensive pregnant women. As it was a comparative study, all the gestational parameters were comparable in the preeclamptic and normotensive groups (Table 3).

**Table 3:** Sociodemographic characteristics of study participants at UGCSH, Northwest Ethiopia, 2022 (*n* = 128).

**Variables**	**Category**	**Preeclamptic women,** **n** **(%)**	**Apparently healthy normotensive pregnant women; n (%)**	**p**
Age (mean ± SD)	N/A	26.81 ± 4.22	26.63 ± 4.23	0.802
Age category	< 35	60 (93.75)	62 (96.88)	
	≥ 35	4 (6.25)	2 (3.12)	
Marital status	Unmarried	14 (21.9)	7 (10.9)	0.095
	Married	50 (78.1)	57 (89.1)	
Residence	Urban	46 (71.9)	51 (79.7)	0.302
	Rural	18 (28.1)	13 (20.3)	
Educational level	No formal education	15 (23.4)	9 (14.1)	0.41
	Primary education	21 (32.8)	11 (17.2)	
	Secondary education	17 (26.6)	25 (39.0)	
	College and university	11 (17.2)	19 (29.7)	
Occupation	Housewife	22 (34.4)	27 (42.2)	0.235
	Government‐employee	17 (26.6)	12 (18.8)	
	Farmer	16 (25.0)	10 (15.6)	
	Others	9 (14.1)	15 (23.4)	
Monthly income	< 2500	35 (54.7)	31 (48.4)	0.112
	*2500–5000*	*14 (21.9)*	*24 (37.5)*	
	*> 5000*	*15 (23.4)*	*9 (14.1)*	

*Note:* *n*, Number of the participant; occupation “others” refers to tea sellers, students, and daily laborers; N/A, not applicable.

### 3.2. Gestational and Clinical Characteristics of Study Participants

The gestational weeks of pregnant women ranged from 21 weeks to 43 weeks with a higher percentage in the third trimester for both groups (55 [85.94%] for preeclamptic women and 54 [84.38%] for apparently healthy normotensive pregnant women). This study noted that all gestational‐related variables had statistically insignificant differences between preeclamptic women and apparently healthy normotensive pregnant women (*p* > 0.05) (Table 4).

**Table 4:** Gestational characteristics of study participants at UGCSH, Northwest Ethiopia, 2022 (*n* = 128).

**Variables**	**Category**	**Preeclamptic women,** **n** **(%)**	**Apparently healthy normotensive pregnant women,** **n** **(%)**	**p**
Gestational age in weeks	20–36.9	42 (65.6)	50 (78.1)	0.072
	37–41.9	11 (17.2)	11 (17.2)	
	≥ 42	11 (17.2)	3 (4.7)	
Trimester	Second	9 (14.1)	10 (15.6)	0.804
	Third	55 (85.94)	54 (84.38)	
Gravidity	Primigravida	32 (50.0)	26 (40.6)	0.287
	Multigravida	32 (50.0)	38 (59.4)	
Parity	Nulliparous	32 (50.0)	28 (43.4)	0.126
	Multipara	19 (29.7)	29 (45.3)	
	Grand‐multipara	13 (20.3)	7 (10.9)	

Abbreviation: n, Number of the participant.

**Figure 1:**
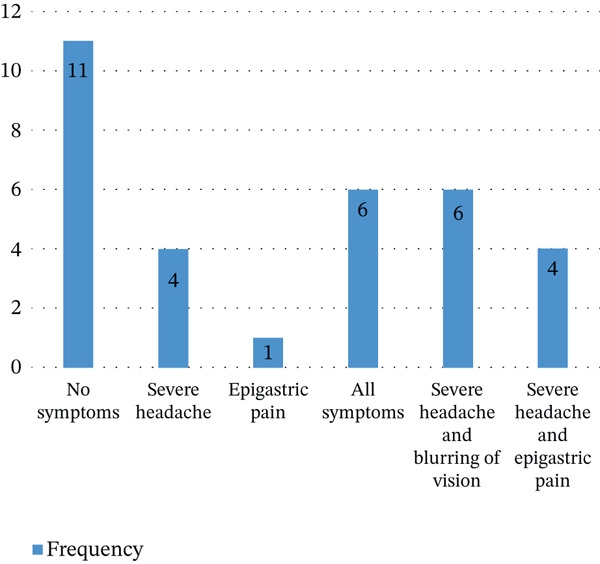
Frequency of severity symptoms among preeclamptic patients with blood pressure ≥160/110 mmHg.

A total of 21 (65.6%) of preeclamptic women with blood pressure ≥ 160/110 mmHg had one or more severe symptoms (Figure 1). The mean SBP of the preeclamptic women was 155.39 ± 11.45 mmHg. Apparently healthy normotensive pregnant women had a mean SBP of 108.67 ± 12.70 mmHg.

### 3.3. Assessment of Biochemical Parameters Among Apparently Healthy Normotensive Pregnant Women and Preeclamptic Women

There was a statistically significant difference between the mean serum levels of LDH, calcium, magnesium, and potassium but there was a statistically insignificant difference in mean sodium levels between preeclamptic and apparently healthy normotensive pregnant women (Table 5).

**Table 5:** Independent *t*‐test comparison of serum LDH and electrolyte levels of preeclamptic women and apparently healthy normotensive pregnant women at UOGCSH, Northwest Ethiopia, 2022 (*n* = 128).

**Variables**	**M** **e** **a** **n** ± **S** **D**		**p**
	**Preeclamptic women**	**Apparently healthy normotensive pregnant women**	
LDH (U/L)	449.86 ± 89.10	259.5 ± 49.32	< 0.001∗
Magnesium (mg/dl)	1.57 ± 0.24	1.72 ± 0.16	< 0.001∗
Calcium (mg/dl)	8.52 ± 0.63	9.23 ± 04113	< 0.001∗
Sodium (mmol/l)	135.89 ± 1.99	136 ± 1.95	0.754
Potassium (mmol/l)	3.79 ± 0.42	3.94 ± 0.35	0.029∗

**Abbreviations:** LDH, lactate dehydrogenase; SD, standard deviation; UOGCSH, University of Gondar Comprehensive Specialized Hospital.

∗ statistically significant at *p* < 0.05.

Trimester‐wise analysis showed that elevated LDH, hypomagnesemia, hypocalcemia, and hypokalemia were more frequent among preeclamptic women, particularly in the third trimester. Hypermagnesemia was rare and no cases of hypernatremia or hyponatremia were observed in either group (Table 6).

**Table 6:** Trimester‐wise distribution of abnormal biochemical parameters.

**Abnormal biochemical parameters**	**Study groups**	**Trimester**		
		**Second trimester** **(** **P** **E** = 9, **N** **T** = 10**)**	**Third trimester** **(** **P** **E** = 55, **N** **T** = 54**)**	**Total**
Elevated LDH	Preeclamptic	3 (33.33%)	12 (21.82%)	15
	Normotensive	0 (0%)	0 (0%)	0
Hypomagnesemia	Preeclamptic	1 (11.11%)	3 (5.46%)	4
	Normotensive	0 (0%)	1 (1.82%)	1
Hypocalcemia	Preeclamptic	1 (11.11%)	17 (30.91%)	18
	Normotensive	0 (0%)	0 (0%)	0
Hyponatremia	Preeclamptic	0 (0%)	0 (0%)	0
	Normotensive	0 (0%)	0 (0%)	0
Hypokalemia	Preeclamptic	1 (11.11%)	10 (18.18%)	11
	Normotensive	0 (0%)	1 (1.82%)	1

**Abbreviations:** NT, apparently healthy normotensive pregnant women; PE, preeclamptic women.

### 3.4. Comparison of Mean LDH and Electrolyte Levels Between Apparently Healthy Normotensive Pregnant Women, Preeclampsia Without Severe Features, and Preeclampsia With Severe Features

Majority of the parameters under study were significantly affected by severity of preeclampsia. Serum LDH was much higher and serum calcium and potassium were significantly lower in cases with preeclampsia with severe features as compared with those without severe preeclampsia. Magnesium showed insignificant decrement in preeclampsia with severe features than without severe features. Sodium values were comparable and did not show a significant difference between the women with or without severe features (Table 7).

**Table 7:** One‐way ANOVA analysis of biochemical test parameters of apparently healthy normotensive pregnant women, preeclampsia without severe features, and preeclampsia with severe features at UOGCSH, 2022 (*n* = 128).

**Parameter**	**Mean ± SD**			**F** **-value**	**p**
	**Apparently healthy normotensive pregnant women**	**Preeclampsia without severe feature**	**Preeclampsia with severe feature**		
LDH (U/L)	259.5 ± 49.32^∗∗∗^^b,^^∗∗^^c^	379.75 ± 57.22^∗∗∗^^a,^^∗∗∗^^c^	519.97 ± 52.07^∗∗∗^^a,^^∗∗∗^^b^	278.707	< 0.001 ^∗^
Magnesium (mg/dl)	1.72 ± 0.16^∗^^b,^^∗∗∗^^c^	1.60 ± 0.25^∗^^a^	1.54 ± 0.23^∗∗∗^^a^	10.199	< 0.001 ^∗∗∗^
Calcium (mg/dl)	9.23 ± 0.41^∗∗^^b,^^∗∗∗^^c^	8.82 ± 0.53^∗∗^^a,^^∗∗∗^^c^	8.22 ± 0.58^∗∗∗^^a,^^∗∗∗^^b^	45.168	< 0.001 ^∗∗∗^
Sodium (mmol/l)	136 ± 1.95	136.31 ± 2.01	135.47 ± 1.90	1.543	0.218
Potassium (mmol/l)	3.94 ± 0.35^∗∗^^c^	3.91 ± 0.39^∗^^c^	3.67 ± 0.42^∗∗^^a,^^∗^^b^	5.728	0.004 ^∗∗^

**Abbreviations:** ANOVA, analysis of variance; LDH, lactate dehydrogenase.

^a^ ^a^significantly differed when compared with apparently healthy normotensive pregnant women.

^b^ ^b^significantly differed (through Bonferroni post hoc test) when compared with preeclampsia without severe features.

^c^ ^c^significantly differed when compared with preeclampsia with severe features.

∗statistically significant at *p* < 0.05, ∗∗significant at *p* < 0.01, ∗∗∗significant at *p* < 0.001.

### 3.5. Assessment of the Potential Diagnostic Value of Test Parameters for Preeclampsia

**Figure 2:**
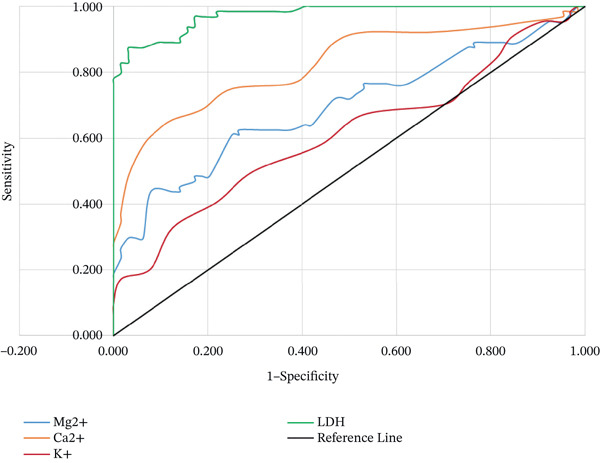
Receiver operator characteristic curve of serum LDH, magnesium, calcium, and potassium with preeclampsia incidence.

Figure 2 shows the ROC curves for serum LDH, magnesium, calcium, and potassium in identifying preeclampsia, with the AUC values indicating the overall diagnostic accuracy of each parameter. The AUC for LDH was 97.5% (*p* < 0.001), with an optimal cutoff of 350 U/L yielding sensitivity of 87.5% and specificity of 96.9%, indicating excellent discriminatory ability. Serum magnesium showed a lower AUC of 69.5% (*p* < 0.001) with a cutoff of 1.505 mg/dl (sensitivity 43.8% and specificity 92.2%), suggesting poor diagnostic potential. For serum calcium, the AUC was 0.823 (*p* < 0.001), demonstrating very good discrimination; cutoff values of 8.75 mg/dl (sensitivity 65.6% and specificity 87.5%) and 8.65 mg/dl (sensitivity 62.5% and specificity 90.6%) indicated that lower calcium levels are associated with preeclampsia. Serum potassium had the lowest AUC of 0.61 (*p* = 0.032) with cutoffs of 3.5 mmol/L (sensitivity 32.8% and specificity 87.5%) and 3.75 mmol/l (sensitivity 50% and specificity 70.3%), reflecting poor discriminative capacity.

## 4. Discussion

The gestational and demographic parameters were comparable in the normotensive and preeclamptic women to avoid any influence of these variables on various study parameters. The present study demonstrated significantly higher mean serum LDH levels in preeclamptic women compared with apparently healthy normotensive pregnant controls, and a stepwise increase with disease severity (*p* < 0.001). Our findings are consistent with previous studies that compared 70 preeclamptic with 35 normotensive women, and 30 preeclamptic with 30 normotensive women [23, 24]. These findings support the established view that LDH serves as a marker of cellular injury, tissue hypoxia, and endothelial dysfunction—central processes in preeclampsia. Elevated LDH also reflects widespread cellular necrosis in organs such as the liver, kidney, lung, and placenta [25]. However, some studies did not identify statistically significant changes between LDH levels of apparently healthy normotensive pregnant women and preeclamptic women [26, 27]. The possible justification might be due to the difference in a gestational week of preeclamptic women and apparently healthy normotensive pregnant women. In our study, the mean ± SD gestational week of preeclamptic patients was insignificantly higher (33.98 ± 5.80 weeks) than that of apparently healthy normotensive pregnant women (32.95 ± 4.31 weeks). However, in study done in Zahedan, Iran, the gestational week of apparently healthy normotensive pregnant women (38 ± 1.4 weeks) was significantly higher in preeclamptic women (36.16 ± 3.4 weeks) [27]. So, the level of serum LDH may be increased in apparently healthy normotensive pregnant women in response to the normal metabolic demands of the growing fetus [28].

In the present study, the diagnostic value of LDH in diagnosing the occurrence of preeclampsia was excellent (AUC = 97.5*%*) with a sensitivity of 87.5% and specificity of 96.9% at the optimum cutoff value of 350 U/L. In this study, serum LDH demonstrated better diagnostic accuracy than reported by Asadi et al. [29], who found an AUC of 80.5% with 89.62% sensitivity and 59.3% specificity at a cutoff of 336 U/L. The possible reason for the variation in the optimum cutoff point of LDH could be the difference in the type of chemistry analyzer, methodology, and sample processing used for LDH determination. However, a meta‐analysis by Pergialiotis et al. [30] on serum LDH values in hypertensive disorders of pregnancy concluded that evidence regarding the diagnostic role of LDH for preeclampsia in clinical practice remains insufficient.

In our study, serum magnesium level was found to be significantly lower (*p* < 0.001) in preeclamptic women compared with apparently healthy normotensive pregnant women. There was also a reduction in the mean levels of serum magnesium between preeclampsia without the severe feature and preeclampsia with severe features but a statistically insignificant difference through post hoc analysis of one‐way ANOVA (*p* = 0.683). The result obtained in the present study is in line with previous studies that have demonstrated a significant decrement in serum levels of magnesium in preeclampsia [31, 32]. This supports the hypothesis that magnesium deficiency may play a contributory role in the pathophysiology of preeclampsia. The clinical efficacy of magnesium sulfate in preventing recurrent seizures in eclampsia further suggests that magnesium homeostasis is altered in this condition [33]. The normal serum magnesium levels in pregnant adults are generally 1.5–2.2 mg/dl depending on the trimester. Major obstetric authorities and guideline bodies including WHO, Society of Obstetricians and Gynecologists of Canada (SOGC), and the American College of Obstetricians and Gynecologists, emphasize the therapeutic role of magnesium sulfate for seizure prophylaxis and treatment in severe preeclampsia and eclampsia, but do not recommend routine measurement of serum magnesium as a diagnostic or screening test for preeclampsia. Instead, magnesium sulfate is recommended as first‐line therapy for prevention and treatment of eclampsia where indicated [34]. Meta‐analyses and systematic reviews report that, on average, circulating magnesium concentrations are lower in women with preeclampsia compared with normotensive pregnant controls, supporting an association between lower serum magnesium and the disease [12]. In contrast to our study, some researchers did not find a statistically significant difference in serum magnesium levels between preeclamptic women and apparently healthy normotensive pregnant women. For instance, Owusu Darkwa et al. conducted a comparative study at the Korle‐Bu Teaching Hospital, Ghana, involving 30 preeclamptic women and 30 normotensive pregnant women, and reported no significant difference in serum magnesium levels between the groups. Similarly, Roy et al. studied 50 preeclamptic and 50 normotensive pregnant women in Bangladesh and observed no significant variation in serum magnesium levels [35, 36]. This discrepancy may be due to the variation in the study population, disease severity, and dietary intake. Our findings are also not in agreement with studies from Iran and Nigeria that reported no significant difference in serum magnesium levels between preeclamptic and normotensive pregnant women. Abedzadeh et al. conducted a study in Kerman province of Iran involving 40 preeclamptic and 40 normotensive pregnant women and found comparable serum magnesium concentrations between the two groups [37]. In addition, Okoror et al. studied 27 preeclamptic and 54 normotensive pregnant women in Nigeria and also observed no statistically significant variation in serum magnesium levels [38]. These findings suggest that hypomagnesemia may not be a universal biochemical feature of preeclampsia, and the inconsistency across studies could be explained by population‐specific dietary intake, nutritional supplementation, genetic predisposition, or differences in laboratory assay methodologies.

In this study, serum magnesium showed moderate diagnostic accuracy for preeclampsia (AUC = 69.5*%*, *p* < 0.001) at a cutoff ≤ 1.505 mg/dl, with high specificity (92.2%) but low sensitivity (43.8%). This indicates that although reduced magnesium levels are fairly specific to preeclampsia, they lack sensitivity, suggesting that hypomagnesemia may accompany the disorder but is not a reliable standalone diagnostic marker. A study conducted in Novi Sad, Serbia, indicated that a concentration cutoff level at ≤ 0.81 mmol/L (1.97 mg/dl) had a sensitivity of 77.0% and specificity of 71.6% with AUC 79.4% and *p* < 0.001 [39]. Overall, these findings indicate that reduced serum magnesium in preeclampsia likely reflects secondary physiological changes, rather than a primary diagnostic feature.

In the current study, we found significantly lower serum calcium levels in preeclamptic women than in normotensive controls (*p* < 0.001), with further reduction observed in cases with severe features compared with those without severe features (*p* < 0.001). In line with our findings, different researchers also report a significant decrease in serum calcium levels compared with apparently healthy normotensive pregnant women. For example, Akhtar et.al. conducted a study involving 60 preeclamptic and 30 normotensive pregnant women, whereas Ephraim et al. involved 120 women with pregnancy induced hypertension, 100 women with preeclampsia and 160 healthy, age‐matched pregnant women; both found significantly lower calcium levels in the preeclamptic group [40, 41]. A systematic review and meta‐analysis by Eslamzadeh et al. concluded that circulating calcium levels are significantly lower in women with preeclampsia than in healthy pregnant controls [12].

The reduced serum calcium in preeclampsia from our study and others may be explained by a dietary deficiency of calcium during pregnancy due to feto‐maternal requirements. Hence, low dietary calcium intake has been associated with elevated parathyroid hormone and renin activity, which increase intracellular calcium in vascular smooth muscle cells, thereby promoting vasoconstriction and hypertension [42]. However, other studies demonstrated an insignificant serum calcium difference between preeclamptic women and apparently healthy normotensive pregnant women [35, 43]. These findings may be explained following the observation that preeclamptic women have a decreased level of daily urinary calcium excretion, fractional excretion of calcium, and significantly reduced 1, 25‐dihydroxycholecalciferol compared to normotensive pregnant women [44]. The variation may be due to a difference in nutritional habits, the severity of the disease, and study participants.

In our study, the ROC curve analysis of serum calcium identified two clinically relevant cutoff values—8.75 mg/dl (sensitivity 65.6% and specificity 87.5%) and 8.65 mg/dl (sensitivity 62.5% and specificity 90.6%)—reflecting a balance between detecting more preeclampsia cases and reducing false positives. These findings indicate that lower calcium levels are strongly associated with preeclampsia and may support its clinical evaluation. The ROC analysis in Indonesia indicated an AUC of 58.3% (*p* = 0.025) with a cutoff value of 4.65 mg/dl (sensitivity 60.2% and specificity 52.9%), suggesting that patients with serum calcium levels below 4.65 mg/dl were more likely to have preeclampsia compared to those with levels above this threshold, with an overall accuracy of 58.3% (*p* = 0.001) [45]. Another study done in China revealed that calcium‐identified preeclampsia with AUC 67% (sensitivity 67% and specificity 39%) at a cutoff level of less than 1.89 mmol/l [46]. The discrepancy in the chemistry analyzer could cause the variation of cutoff values and specific analyzers tend to exaggerate some metrics more than others [47]. The other cause for difference could be due to imbalanced or small sample size that can skew AUC estimates.

In the present study, the mean sodium level in preeclamptic women (135.89 ± 1.99 mmol/l) was slightly lower than in apparently healthy normotensive pregnant women (136 ± 1.95 mmol/l); however, the difference was not statistically significant (*p* = 0.754). Similarly, one‐way ANOVA analysis revealed no significant difference among the groups (*p* = 0.218). Our findings differ from those of a study conducted in Pakistan [18], where a larger sample size was used (160 preeclamptic and 74 normotensive women). Our finding also contradict the reports of a significant reduction in mean serum sodium levels in 30 preeclamptic women as compared with 30 apparently healthy normotensive pregnant women in Ghana [48]. Other studies have also revealed statistically significant elevated serum sodium levels in preeclamptic women compared to apparently healthy normotensive pregnant women [49, 50]. These discrepancies suggest that the role of serum sodium in the pathophysiology of preeclampsia remains unclear, likely due to population differences and methodological variations among studies. Although sodium handling abnormalities have been hypothesized due to altered renin–angiotensin–aldosterone activity, recent evidence does not support sodium restriction as a preventive or therapeutic strategy for preeclampsia [17].

In the current study, preeclamptic women showed a statistically significant reduction (*p* = 0.029) in mean serum potassium levels compared to apparently healthy normotensive pregnant women. The current study showed a significantly lower level (0.021) in preeclampsia with severe features than in preeclampsia without severe features. Similar observations were made in different studies in India and Ghana [19, 48]. It can also be characterized due to the role of potassium in vascular smooth muscle and endothelial cells regulating calcium entry into the cells, resulting in vasodilatation, although the mechanism remains unclear [51]. In contrast, another study showed statistically insignificant difference in the serum potassium level between preeclamptic women and apparently healthy normotensive pregnant women [43]. The difference obtained in different studies may be due to the variation in the study population and dietary intake. Therefore, due to these contrasting results, further large‐scale, multicenter studies are warranted to clarify the exact relationship between potassium levels and the development and progression of preeclampsia. The low AUC (0.61) from our ROC analysis indicates poor diagnostic value, suggesting that potassium changes are secondary rather than causative. The cutoff values were 3.5 (sensitivity 32.8% and specificity 87.5%) and 3.75 mmol/l (sensitivity 50% and specificity 70.3%). These findings show that serum potassium levels are not the preferred tool for diagnosing the condition.

### 4.1. Conclusion and Recommendations

The values of LDH were higher and those of serum calcium, magnesium and potassium were significantly lower in women with preeclampsia as compared with normotensive pregnant controls. Calcium and potassium values were significantly affected with increasing severity of preeclampsia. Magnesium showed significant decrement in preeclampsia, although insignificant reduction was noted in preeclampsia with severe features as compared with without sever features. On the other hand, values of serum sodium were comparable in normotensive and preeclamptic women and did not show any variation with increasing severity of preeclampsia.

So, this study helps the utilization of serum LDH and specific electrolytes as biochemical markers of preeclampsia severity. Integrating these indicators into clinical practice could lead to earlier identification, better risk assessment, and more informed decision‐making, ultimately better maternal and fetal care in preeclampsia management. Therefore, it is better to focus on measuring serum LDH and electrolytes to detect early or for better treatment of preeclampsia patients.

**Nomenclature**
ANOVA: analysis of variance
DBP: Diastolic blood pressure
LDH: lactate dehydrogenase
ROC: receiver operator characteristics.
SBP: Systolic blood pressure
UGCSH: University of Gondar Comprehensive Specialized Hospital
U/L: unit per liter

## Ethics Statement

Ethical clearance with reference number 770/8/2021 was obtained from the Institutional Review Committee of the University of Gondar, and a letter of cooperation was obtained from the University of Gondar Comprehensive Specialized Hospital medical director′s office before the actual data collection started. Written informed consent was obtained from the study participants. Participants had a full right to continue or withdraw from the study. Privacy and confidentiality of information were appropriately kept and their names were not recorded.

## Disclosure

All authors read and approved the final manuscript.

## Conflicts of Interest

The authors declare no conflicts of interest.

## Author Contributions

E.Y.T. conceived and designed the study, took part in methodology, data collection, software, formal analysis, resources, data curation, writing—original manuscript draft, writing—review & editing, visualization, and supervision. M.D.M participated in visualization, methodology, software, data curation, analysis, reviewing the manuscript draft, and supervision. M.F.Z., H.T.E., F.S.T., and M.J. took part in methodology, software, data curation, analysis, reviewing the manuscript draft, and supervision.

## Funding

No funding was received for this manuscript.

## Data Availability

The datasets used and/or analyzed during the current study available from the corresponding author on reasonable request.
